# A cost-effective, machine learning-driven approach for screening arterial functional aging in a large-scale Chinese population

**DOI:** 10.3389/fpubh.2024.1365479

**Published:** 2024-03-20

**Authors:** Rujia Miao, Qian Dong, Xuelian Liu, Yingying Chen, Jiangang Wang, Jianwen Chen

**Affiliations:** ^1^Health Management Medicine Center, The Third Xiangya Hospital, Central South University, Changsha, China; ^2^School of Science, Hunan University of Technology and Business, Changsha, China

**Keywords:** machine learning, XGBoost, arterial stiffness, physical examination, questionnaire, feature

## Abstract

**Introduction:**

An easily accessible and cost-free machine learning model based on prior probabilities of vascular aging enables an application to pinpoint high-risk populations before physical checks and optimize healthcare investment.

**Methods:**

A dataset containing questionnaire responses and physical measurement parameters from 77,134 adults was extracted from the electronic records of the Health Management Center at the Third Xiangya Hospital. The least absolute shrinkage and selection operator and recursive feature elimination-Lightweight Gradient Elevator were employed to select features from a pool of potential covariates. The participants were randomly divided into training (70%) and test cohorts (30%). Four machine learning algorithms were applied to build the screening models for elevated arterial stiffness (EAS), and the performance of models was evaluated by calculating the area under the receiver operating characteristic curve (AUC), sensitivity, specificity, and accuracy.

**Results:**

Fourteen easily accessible features were selected to construct the model, including “systolic blood pressure” (SBP), “age,” “waist circumference,” “history of hypertension,” “sex,” “exercise,” “awareness of normal blood pressure,” “eat fruit,” “work intensity,” “drink milk,” “eat bean products,” “smoking,” “alcohol consumption,” and “Irritableness.” The extreme gradient boosting (XGBoost) model outperformed the other three models, achieving AUC values of 0.8722 and 0.8710 in the training and test sets, respectively. The most important five features are SBP, age, waist, history of hypertension, and sex.

**Conclusion:**

The XGBoost model ideally assesses the prior probability of the current EAS in the general population. The integration of the model into primary care facilities has the potential to lower medical expenses and enhance the management of arterial aging.

## 1 Introduction

Vascular aging, regardless of the presence of atherosclerosis, is characterized by intimal and medial thickening and a loss of arterial elasticity, leading to vascular stiffness (1, 2). Population-based studies indicated that vascular aging should no longer be only considered a part of normal aging but rather influenced by industrialized lifestyle (3) and increased with the level of urbanization (4). Aortic pulse wave velocity (PWV) is considered a physiological method for quantifying arterial functional aging (5, 6) and serves as a surrogate marker of arterial stiffness, which is strongly related to cardiovascular diseases (CVDs) morbidity (7, 8).

Approximately 35% of individuals aged less than 40 years present a PWV value that was above the 90th percentile of PWV expected for their age, according to the European Reference Values Collaboration (9), and widespread PWV screening remains lacking in practice (10). Vascular aging is strongly influenced by acquired risk factors, primarily related to lifestyle choices and cardio-metabolic indicators such as smoking (11, 12), high blood pressure (13, 14), and glucose levels (15). This implies that arterial aging, as measured by PWV, can be preemptively evaluated with the aid of a suitable algorithm. This might allow for the precise identification of high-risk individuals at minimal or even zero cost, optimizing the efficiency of screening initiatives and reducing the health investment required for the vast low-risk population. We hereby reintroduce the concept of pretest probability. For example, in clinical practice, before diagnosing coronary heart disease, it is necessary to assess the disease’s prior probability based on symptoms and other factors. This assessment informs the next steps in patient testing (16). Similar to this scope, our testing employs cross-sectional big data and machine learning methods to determine the pretest probability of current vascular aging.

Machine learning (ML) has been successfully employed in medicine to establish and develop accurate models (17). It outperforms conventional statistical methods by automatically training itself and improving its performance without the need for intricate programming. ML has the capacity to learn from diverse data modules and model complex relationships, resulting in more accurate predictions (18). A gradient boosting-based model for arterial stiffness assessment using clinical characteristics was constructed in a cohort of 1,672 patients with diabetes (19), demonstrating effective classification for elevated arterial stiffness (EAS) within this specific group. Based on our speculation that the prevalence of elevated PWV would be higher in individuals with diabetes, and considering the distinct parameters and contributing features of the model in this population, we realized that the model for the general population would likely differ substantially.

By leveraging a vast dataset gathered from a substantial cohort of physical examinees, encompassing Pulse Wave Velocity (PWV) metrics, along with detailed insights into lifestyle patterns and fundamental clinical attributes, we employed machine learning (ML) techniques to craft a streamlined and economical screening model for arterial stiffness in the general populace. This approach enables more precise pinpointing of high-risk demographics. Such a strategy may significantly reduce the likelihood of overlooking potential diagnoses and curtail medical squandering, enhancing the overall efficiency and effectiveness of health screenings for arterial aging.

## 2 Methods

### 2.1 Study population and data source

The retrospective dataset was extracted from the electronic healthcare records of the health management center of the Third Xiangya Hospital. The records collected spanned from 2015 to 2021 and constituted the original set. The dataset consisted of 77,191 physical examinees who underwent a brachial-ankle pulse wave velocity (baPWV) test at the health management center. The exclusion criteria were as follows: (1) individuals who were unable to sign informed consent; (2) patients with a diagnosis of end-stage renal disease and aortopathy. Samples with massive missing data or excessive outliers whose irrationality was justified by the clinician were deleted, resulting in a final dataset of 77,134 individuals. All participants signed the informed consent form. The study was conducted in accordance with the Declaration of Helsinki and approved by the Ethics Committee of the Third Xiangya Hospital (No 2020-S609).

### 2.2 Feature characteristics and definitions

The dataset included a total of 82 features, which encompassed various types of information. These features included physical examination measurements such as body mass index (BMI), waist circumference (WC), and systolic/diastolic blood pressure (SBP/DBP). Laboratory indicators were also present in the dataset and included measurements such as fasting blood sugar (FBS), lipid profile, and renal function markers. Additionally, questionnaire information was collected, including previous diagnoses of diseases, lifestyle factors (diet preference, smoking, alcohol consumption, exercise habits, working and sedentary time, and sleep status), and health literacy regarding basic medical knowledge. For specific definitions within the dataset, alcohol consumption was defined as consuming more than 50 g of alcohol per week. The current smoking status was defined as smoking more than one cigarette per day. The baPWV measurements were obtained using a non-invasive measurement system called VP-2000 manufactured by Colin Co Ltd., Komaki, Japan. Trained medical staff performed the measurements in a room after the subjects had rested in a supine position for 10 min. Four pneumatic cuffs were attached to the bilateral arms and ankles to obtain pulse waves. The baPWV was automatically calculated using the formula (La-Lb)/Tba, where La represents the distance between the heart and ankle, Lb represents the distance between the heart and brachium, and Tba represents the time difference between the initial increase in the brachial waveform and that in the ankle waveform. Elevated arterial stiffness (EAS) was considered as baPWV≥1,400 cm/s (20).

### 2.3 Data processing and statistical analysis

Nineteen features in the original dataset are related to detailed questions about smoking, drinking, exercising, and work intensity, including types and frequency. To reduce the redundancy of the data, these data are not included in the analysis for now. If factors such as smoking and drinking prove important in later analyses, this part of the data will be specifically analyzed. However, since factors such as smoking and drinking were not considered highly important in subsequent analyses, no further specific analysis was conducted on these questions. If a sample has more than 30% of its variables missing, then the sample is deleted. Therefore, a total of 57 samples were deleted. For the remaining missing data, single-value imputation was used to handle features. Ultimately, 63 features and 77,134 samples were retained. The entire dataset was randomly divided into a training set (70%, 53,993 samples) and a test set (30%, 23,141 samples). In the test set, there were 13,600 samples for the non-EAS category and 9,541 samples for the EAS category.

Continuous measurement data conforming to the normal distribution were expressed as mean ± standard deviation, otherwise, the quartile was adopted, and the difference between categorical features was calculated using the chi-squared test. The difference of *p* < 0.05 on both sides was considered statistically significant.

### 2.4 Feature selection

In the field of machine learning, feature selection can eliminate irrelevant or redundant features, thereby reducing the number of features, improving model accuracy, and reducing runtime. Feature selection methods are mainly divided into three categories: filter, embedded, and wrapper methods.

Filter method: Features that did not show significant differences across categories meant they had no use for the prediction target and would be deleted.

Embedded: The features were input into the Lasso model for training after the filter method. The L1 regularization parameters were tuned using 10-fold cross-validation, and the coefficient would be shrunken to zero if their features were not important.

Wrapper: After filtering through Lasso regression, if there were still quite a number of features left, the wrapper method could be used for further selection. The working principle of recursive feature elimination (RFE) is to recursively remove features and build a model on the remaining features (in this case, the Lightweight Gradient Elevator (LGBM) is chosen), thereby determining which combinations of features contribute more significantly to the prediction results. Regarding the choice of the number of features, as we continuously increase the number of features, the AUC score of RFE-LGBM gradually increases and tends to stabilize. When the number of features reaches N, the AUC score reaches its maximum value. Adding more features will not enhance the model’s predictive capability, so ultimately, N features are determined for model building through RFE-LGBM.

Based on the common usage and the situation of data being selected at each step, we ultimately employed these three methods.

### 2.5 Parameter optimization and model evaluation

**Figure 1:**
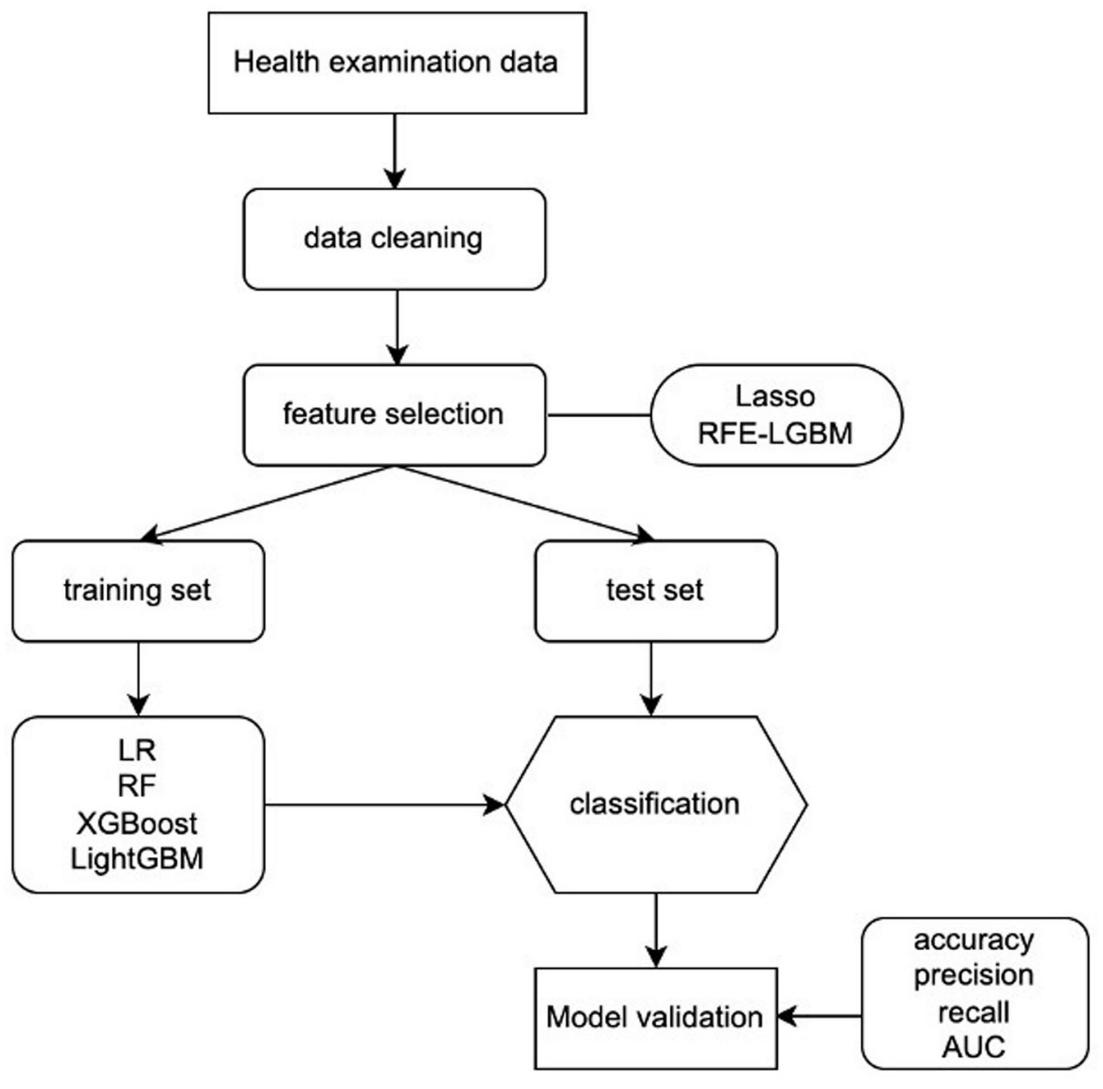
Machine learning flowchart of this study. LR, logistic regression; RF, random forest; XGBoost, extreme gradient boosting; LightGBM, Light gradient boosting machine; RFE-LGBM, recursive feature elimination-Lightweight Gradient Elevator; AUC, area under the receiver operating characteristic curve.

Four machine learning algorithms (logistic regression [LR], random forest [RF], extreme gradient boosting [XGBoost], and light gradient boosting machine [LightGBM]) were used to develop predictive models on the training data. Weights of different classes were assigned by setting parameters in the trained models to deal with data imbalance. The parameters of LR, RF, and LightGBM models were class_weight = “balanced,” and XGBoost’s was scale_pos_weight = “ratio of majority and minority class.” As to the other parameters that required adjustment, grid searching was adopted in the LR model and the Bayesian Optimization Algorithm was applied to the RF, XGBoost, and LightGBM models. To assess the model’s optimization and improve its generalization ability, 10-fold cross-validation was performed. The model’s performance was evaluated by calculating the accuracy, sensitivity, and specificity, and the distinguishing abilities of the risk assessment model were evaluated with the area under the receiver operator characteristic (AUC). The overview of the proposed ML algorithms is shown in Figure 1.

### 2.6 Feature importance ranking

Shapley additive explanations (SHAP) value was defined as the average marginal contribution of a feature value across all possible feature coalitions. It provides insights into the influence of each feature on individual samples, showcasing both positive and negative effects.

ML was implemented in Python (v 3.6) using the sklearn (v. 0.24.1) and XGBoost (v. 1.3.3) packages. Bayesian optimization based on the TPE toolbox (bayes_opt v. 1.2.0) was used to tune hyperparameters for learning algorithms when the best combination of parameters yielded a low model performance. SHAP (V 0.41.0) was for explainable ML.

## 3 Results

### 3.1 Characteristics of the study population

A total of 77,134 subjects were included, with a mean age of 48.6 ± 11.4 years, and 54.9 ± 10.6 years in subjects with EAS. The sample prevalence of EAS was 40.8%. Significant differences (*p* < 0.01) were observed in age, BMI, WC, SBP/DBP, indicators concerning renal function, FBS, total cholesterol (TC), triglyceride (TG), high−/low-density lipoprotein cholesterol (HDL-c/LDL-c) (Table 1), sex, history of cardiovascular diseases, smoking, exercise, work intensity, sedentary time, sleeping quality and time, 16 items of dietary habits, 9 items of negative emotions, and 12 items of health literacy (Table 2) between EAS and non-EAS.

**Table 1:** Features of the participants that are continuous data in primary settings.

Feature	All (*n* = 77,134)	Non-AS (*n* = 45,688)	EAS (*n* = 31,446)	*p* value
Age	48.6 ± 11.4	44.2 ± 9.7	54.9 ± 10.6	<0.001
Body mass index	24.6 ± 3.2	24.2 ± 3.2	25.1 ± 3.1	<0.001
Waist circumference	84.0 ± 9.804	82.3 ± 9.9	86.4 ± 9.1	<0.001
Systolic blood pressure	125.3 ± 16.8	118.0 ± 12.7	135.9 ± 16.3	<0.001
Diastolic blood pressure	77.2 ± 11.5	73.1 ± 9.8	83.1 ± 11.3	<0.001
Blood urea nitrogen	4.9 ± 1.3	4.8 ± 1.2	5.1 ± 1.4	<0.001
Serum creatinine	74.0 ± 18.8	72.8 ± 16.3	75.9 ± 21.9	<0.001
Serum uric acid	347.9 ± 89.6	339.9 ± 89.2	359.6 ± 88.9	<0.001
Fasting blood sugar	5.7 ± 1.5	5.4 ± 1.0	6.1 ± 1.9	<0.001
Total cholesterol	5.1 ± 1.0	5.0 ± 0.9	5.3 ± 1.1	<0.001
Triglyceride	2.0 ± 1.9	1.8 ± 1.7	2.3 ± 2.2	<0.001
High-density lipoprotein cholesterol	1.3 ± 0.3	1.3 ± 0.3	1.3 ± 0.3	<0.001
Low-density lipoprotein cholesterol	2.9 ± 0.9	2.9 ± 0.8	3.0 ± 0.9	<0.001

EAS, elevated arterial stiffness.

**Table 2:** Features of the participants that are categorical data in primary settings.

Features	Chi value	*p*-value	Features	Chi value	*p*-value
Sex	494.529	<0.001	Irritableness	408.974	<0.001
History of hypertension	6905.432	<0.001	Nervousness	523.143	<0.001
History of diabetes	1913.659	<0.001	Anxiousness	263.128	<0.001
History of other CVD	586.573	<0.001	Impatience	306.742	<0.001
Meals on time	828.782	<0.001	Lack of enthusiasm	562.609	<0.001
Eat midnight snack	2184.694	<0.001	Upset	374.698	<0.001
Gluttony	36.759	<0.001	Depression	513.749	<0.001
Dinner party	186.321	<0.001	Difficulty concentrating	378.7	<0.001
Drink milk	526.563	<0.001	Sleep quality	82.116	<0.001
Eating eggs	103.121	<0.001	Sleep duration	264.814	<0.001
Eat beans	13.101	0.001	Active acquisition of medical knowledge	0.2	0.655
Eat fruit	302.005	<0.001	Fasten seat belt	791.405	<0.001
Eat vegetables	415.069	<0.001	Observed stools	114.9	<0.001
Eat meat	100.03	<0.001	Self-measurement of BP/HR	4761.237	<0.001
Eat fatty meat	119.924	<0.001	Take first-aid medicine along	1136.878	<0.001
Eat animal offal	190.865	<0.001	Sunlight exposure	771.451	<0.001
Eat fish	178.008	<0.001	Awareness of normal BP	154.105	<0.001
Drink coffee	487.353	<0.001	Awareness of normal body temperature	13.242	<0.001
Sugary drinks	1276.974	<0.001	Awareness of normal pulse	0.01	0.919
Smoking	207.578	<0.001	Awareness of normal salt intake	79.905	<0.001
Drink alcohol	131.602	<0.001	Awareness of normal BMI	85.353	<0.001
Exercise	81.124	<0.001	Awareness of normal WC	25.583	<0.001
Work intensity	2985.317	<0.001	Awareness of normal FBS	156.494	<0.001
Sedentary duration	173.991	<0.001	Awareness of normal triglyceride	31.048	<0.001
Depressed	587.383	<0.001	Awareness of normal TC	1.525	0.217

BP, blood pressure; HR, heart rate; BMI, body mass index; WC, waist circumference; FBS, fasting blood sugar; TC, total cholesterol.

### 3.2 Feature selection

Three health literacy items (active in the acquisition of medical knowledge and awareness of normal pulse rate/total cholesterol) with no statistical difference were removed from further analysis, and 60 features were input for subsequent feature selection.

**Figure 2:**
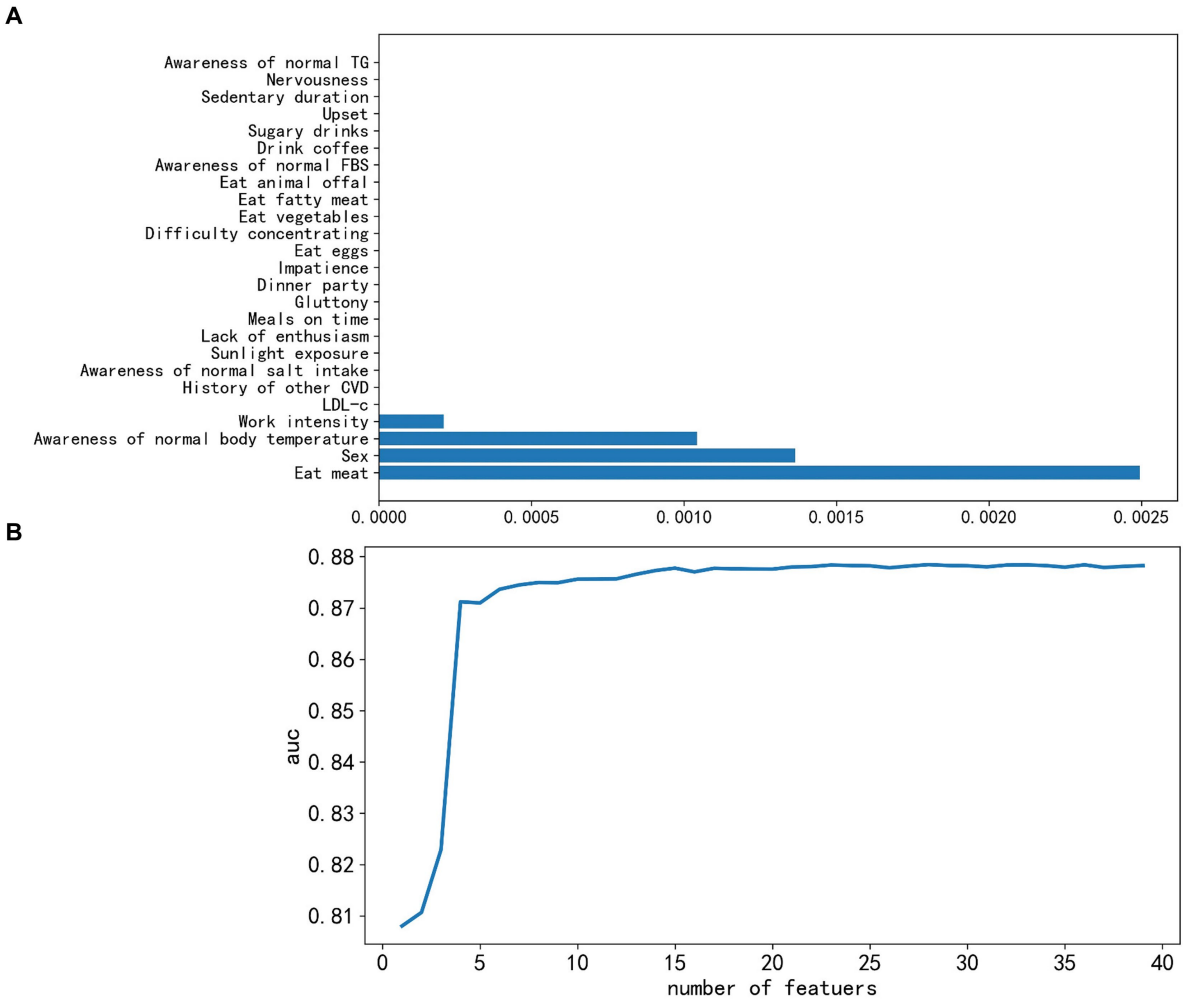
Feature selection successively using the Lasso algorithm and recursive feature elimination-lightweight gradient elevator (RFE-LGBM). **(A)** Coefficients profile of bottom 25 features resulting from the Lasso algorithm, where features with zero coefficients were removed. **(B)** Five-fold cross-validation criterion of RFE based on LGBM, 23 features were optimal and finally kept. AUC, area under the receiver operating characteristic curve.

The Lasso regression ranked the features based on their parameter values, and the bottom 25 features (Figure 2A) were visually observed. Subsequently, 21 features with zero parameter values were deleted, including LDL-c, history of other cardiovascular diseases (excluding hypertension and diabetes), awareness of normal salt intake/FBS/TG values, sunlight exposure, lack of enthusiasm, impatience, difficulty concentrating, nervousness, upset, meals on time, gluttony, frequency of consumption of eggs, vegetables, fat, animal offal, coffee, sugary drinks, sedentary duration, and dinner party.

The 39 reserved features were considered too much, and we adopted the LGBM model to train them further. With each addition, AUC scores for the test set were recorded. As depicted in Figure 2B, there was a discernible decrease in the score as we integrated 23 features one by one; continuous feature addition not only raised computational costs but also failed to enhance model performance. Consequently, we discarded the 16 non-contributory features. The remaining 23 significant features were sex, age, history of hypertension, SBP, DBP, BMI, WC, TC, TG, HDL-c, FBS, serum uric acid, serum creatinine, urea nitrogen, exercise, awareness of normal BP, the consumption frequency of items such as fruit, milk, bean products, alcohol, and smoking, work intensity, and irritableness. An important observation was the strong linear correlation between BMI and WC (*r* = 0.86) and between SBP and DBP (*r* = 0.78) (Supplementary Figure 1). For efficiency, we retained only WC and SBP, leading to a final 21 features for ML evaluation. It is noteworthy that we also developed a model that prioritizes features that are cost-effective and easily obtainable. Therefore, lab indicators were omitted, leaving us with 14 salient features.

### 3.3 Model performance and feature importance ranking

The performance of four classifiers—logistic regression (LR), random forest (RF), XGBoost, and LightGBM—across the training and test sets is detailed in Table 3 (for the 14-feature model) and Supplementary Table 1 (for the 21-feature model). Intriguingly, the incorporation of laboratory indicators in the 21-feature models resulted in only marginal improvements in performance compared to the 14-feature models. For instance, the AUC test scores showed negligible differences: LR (0.8746 vs. 0.8706), RF (0.8697 vs. 0.8695), XGBoost (0.8754 vs. 0.8710), and LGBM (0.8732 vs. 0.8702). This increment came at a significant cost to the testers, suggesting that classifiers with only 14 features are more suitable for broad public applications. In terms of overall performance, the XGBoost model modestly outshone the other classifiers in multiple metrics, including accuracy (0.7878), sensitivity (0.78), specificity (0.73), and AUC (0.8710) on the test set.

**Table 3:** The results of classification algorithms based on 14 costless features.

Model	Accuracy_train	Accuracy_test	Sensitivity	Specificity	AUC_train	AUC_test
LR	0.7839	0.7877	0.78	0.72	0.8678	0.8706
RF	0.7852	0.7845	0.77	0.72	0.8678	0.8685
XGB	0.7870	0.7878	0.78	0.73	0.8722	0.8710
LGBM	0.7967	0.7865	0.77	0.73	0.8702	0.8702

LR, logistic regression; RF, random forest; XGBoost, extreme gradient boosting; LightGBM, Light gradient boosting machine; RFE-LGBM, recursive feature elimination-Lightweight Gradient Elevator; AUC, area under the receiver operating characteristic curve.

**Figure 3:**
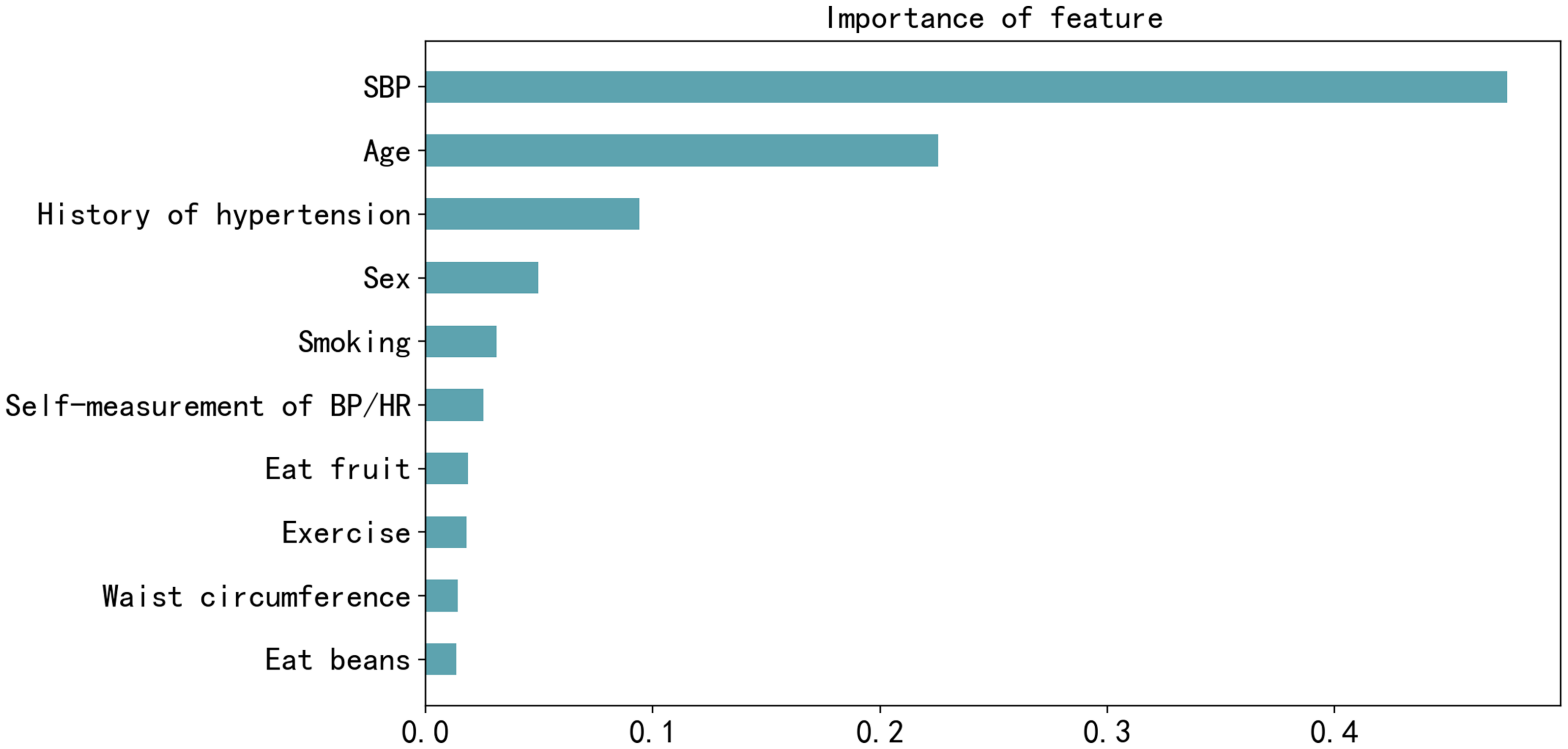
Feature importance plot of the best-performing extreme gradient boosting model measured by F-score. SBP, systolic blood pressure; BP, blood pressure; HR, heart rate.

**Figure 4:**
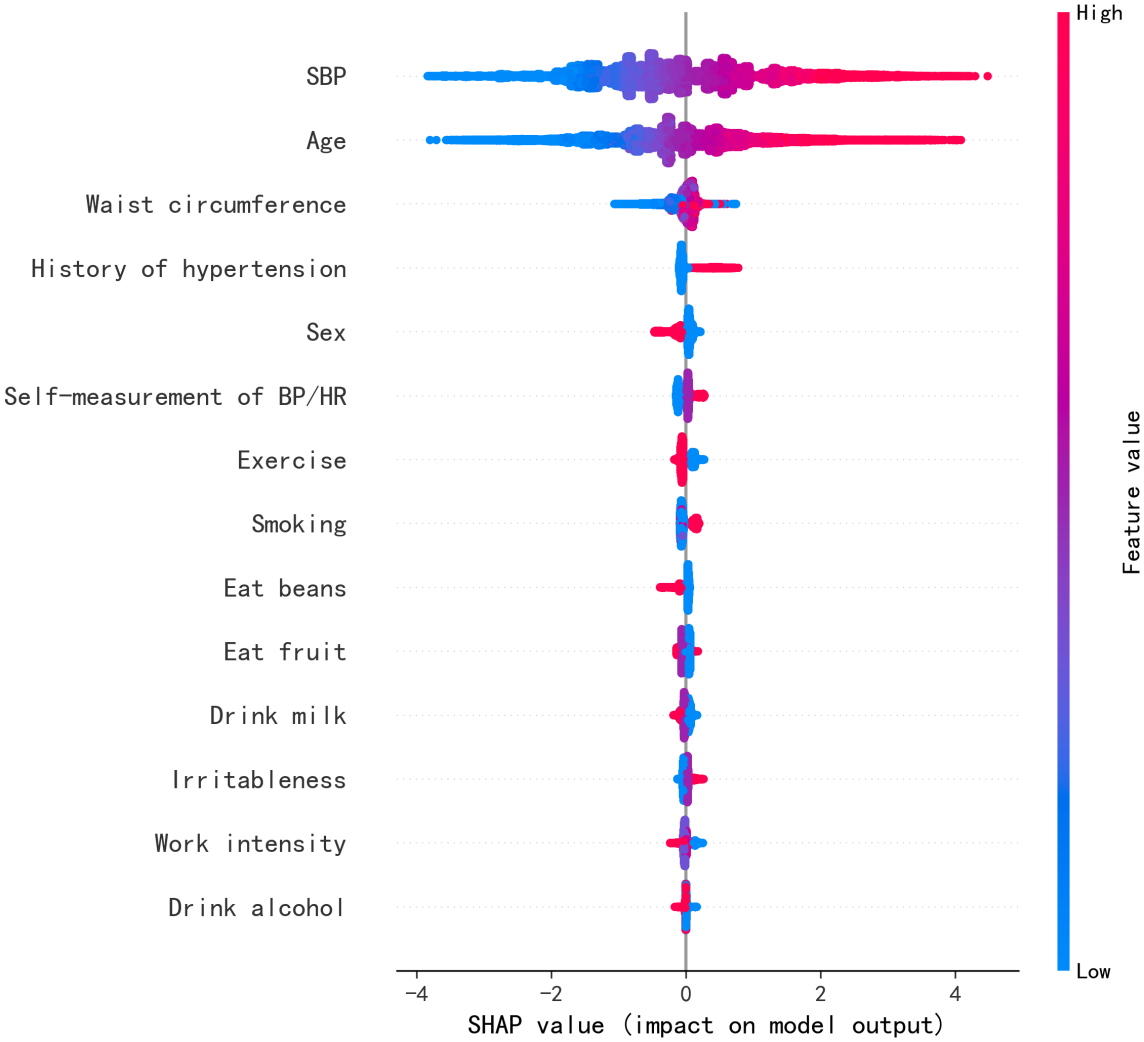
Feature weight sorting by Shapley additive explanations (SHAP) value. SBP, systolic blood pressure; BP, blood pressure; HR, heart rate.

Feature importance was assessed in the XGBoost model, and the top 10 most influential features were identified as SBP, age, history of hypertension, FBS, TG, sex, smoking, fruit consumption, awareness of normal BP, and exercise, as shown in Figure 3. Figure 4 visually presents the SHAP values corresponding to the 14 most important features. The *x*-axis represents the SHAP values, while the *y*-axis lists the features; each dot symbolizes a sample. The color scale varies from low (blue) to high (red), indicating the feature values. A positive SHAP value indicates contributing to EAS formation, whereas a negative value suggests inhibition. The top 5 features in terms of importance were SBP, age, WC, history of hypertension, and sex.

## 4 Discussion

In this study, we established four ML models for arterial stiffness screening in a large-scale physical examination population using easily accessible measurements and questionnaire indicators. Among them, XGBoost demonstrated superior performance in validation. Interestingly, while including optional laboratory features led to a minor enhancement in model performance, the gains may not justify the increased complexity and costs. This model holds promise for predicting early-stage vascular aging, particularly in regions with elevated epidemiological risks.

Traditional CVD risk factors played a noticeable role in affecting arterial stiffness (AS). A meta-analysis (21) highlighted age and blood pressure as the primary determinants of arterial stiffness. Additionally, history of hypertension and waist circumference were also influential factors. Regarding the more costly and invasive laboratory indices, abnormalities in glucose metabolism (22), triglyceride (23), HDL-c, uric acid (24), and glomerular filtration rate (GFR) (25) as determined by serum creatinine have all been linked to varying degrees with arterial stiffness. Our model, which incorporated 21 features—including both questionnaire items and select laboratory indices—aligns well with findings from previous studies. However, one exception was total cholesterol (26, 27), where its potential anti-stiffening effects remain a matter of debate. A notable model for assessing arterial stiffness is the SAGE scoring system. This model, derived from a cohort of 3,943 outpatients and using multiple logistic regression, incorporated variables such as SBP, age, glycemia, and GFR. Impressively, it achieved a 0.77 ROC in its validation cohort (28).

Most traditional models related to cardiovascular risk integrate both laboratory and clinical parameters (29–31). Securing these variables often entails financial expenditures, making the broad application of such models somewhat limited in the general population (32). In our earlier exploration, we compared results derived from both self-assessment items and blood tests with those garnered exclusively from self-assessment features. Intriguingly, the differences were minimal, which we attribute primarily to the overwhelming influence of factors such as blood pressure, age, and waist circumference. A notable strength of our study is its reliance on non-invasive, easily obtainable indicators that come at a minimal cost, which are sufficient to craft a scalable EAS model. However, using a vast number of these indicators poses challenges. These raw datasets tend to be non-linear, intricate, and possibly inter-correlated or influenced by a plethora of confounding variables (33). This complexity could lead to significant deviations and diminished accuracy when building models. Given this backdrop, ML algorithms emerge as a preferable approach, offering a remedy to these pitfalls. We initially employed LASSO and RFE-LGBM to filter out superfluous features and avert overfitting caused by collinearity. Ultimately, leveraging four distinct ML algorithms to assess EAS proved more effective than relying on a single model. Notably, XGBoost marginally surpassed the other three in metrics such as accuracy, sensitivity, specificity, and AUC.

Contrary to conventional methodologies such as logistic regression, which operate under the presumption of variables being independent and purely linear (34), XGBoost adopts a non-parametric approach. It integrates a regularized loss function and marries gradient-boosting algorithms with decision trees, preserving inter-feature correlations (35). We theorize that this unique characteristic underpins XGBoost’s standout performance.

Another study that focused on assessing the risk of EAS in diabetic patients reported ROC values of 0.928 and 0.821 using a gradient-boosting algorithm for a discovery dataset of 760 Chinese individuals and a validation dataset of 912 Japanese individuals, respectively (19). In comparison, our study’s significantly larger dataset ensured a more normal distribution and, consequently, more precise analyses. The ROC values in our study were 0.8826 and 0.8754 for the training and test datasets, respectively. The closer alignment of these values suggests a superior model fit (36). Furthermore, by targeting the general population rather than specialized groups, our model may boast broader applicability.

Currently, PWV assessment is used for patients suspected or diagnosed with CVD and individuals undergoing self-funded health examinations in Chinese hospitals. However, it is challenging to implement PWV screening for the general population outside of hospital settings to enhance the management of CVD. Moreover, the subject’s general physical examination reveals the need for a more standardized approach in developing personalized medical examination programs, particularly concerning vessel checks. Currently, the formulation of these programs is largely influenced by the individual’s economic status, resulting in either missed diagnoses due to inadequate testing or unnecessary resource wastage through over-testing. For instance, the number of physical examinees in China surged from 444 to 549 million in 3 years, according to the China Health Statistics Yearbook, with the average cost per examination amounting to 755.8¥ (37). This typically includes visceral color ultrasound and selected blood tests such as lipid, glucose, hepatic and renal function, and complete blood count (38). However, this cost does not necessarily cover more specialized, expensive tests such as arterial stiffness assessments, CT scans, or gastrointestinal endoscopies. This suggests that early identification measures in primary care remain inadequate, even for those who undergo routine physicals, let alone those who have not visited a hospital. Therefore, incorporating a convenient and easily accessible assessment model into primary care could offer a cost-free initial screening for the general population. This would allow healthcare providers to tailor preventive strategies to those at higher risk. Machine learning models serve as a viable pathway for achieving this, particularly for asymptomatic individuals. At the current stage, the process of transforming a model derived from the research process into a practical clinical tool involves a productization process. This includes placing the model in the cloud or making it into a web-based version, accessible from anywhere. It also involves connecting certain data links, allowing data to be transferred from medical devices to the servers that host the model. We plan to make this model into an easily accessible web-based version.

### 4.1 Limitations

Our study is not free from limitations. First, given its cross-sectional design, it is not possible to infer causal relationships from the data. However, previous studies had established definite causality that we could follow. Our initial intention for the research was to assess the current status of arterial aging, thus making the study design reasonable. Second, the standards for evaluating certain lifestyle factors, such as fruit and bean consumption or exercise habits, were not unified and objectively quantified. This could potentially decrease the prediction’s precision. Finally, since the study relies on data from physical examinations conducted in China, there may be restrictions on the applicability of the results to other ethnicities or cultures.

## 5 Conclusion

The primary aim of our research was to provide an effective model for evaluating the risk of early Arterial Stiffening (EAS) using only questionnaires and physical measurements. Overall, we found that the model’s diagnostic accuracy and assessment capabilities were satisfactory. Incorporating this risk assessment model into primary healthcare settings could significantly improve the prevention and management of arterial aging across the broader populace.

## Data availability statement

The raw data supporting the conclusions of this article will be made available by the authors, without undue reservation.

## Ethics statement

The studies involving humans were approved by the Ethics Committee of the Third Xiangya Hospital. The studies were conducted in accordance with the local legislation and institutional requirements. The participants provided their written informed consent to participate in this study.

## Author contributions

RM: Conceptualization, Funding acquisition, Writing – original draft, Writing – review & editing. QD: Formal analysis, Writing – review & editing. XL: Data curation, Investigation, Writing – review & editing. YC: Data curation, Investigation, Writing – review & editing. JW: Project administration, Supervision, Writing – review & editing. JC: Conceptualization, Formal analysis, Methodology, Writing – review & editing.
