# Supplementary figures and images for: A cost-effective, machine learning-driven approach for screening arterial functional aging in a large-scale Chinese population
Source: Front Public Health. 2024 Mar 20;12:1365479. doi: 10.3389/fpubh.2024.1365479 (PMC10987946; doi:10.3389/fpubh.2024.1365479)

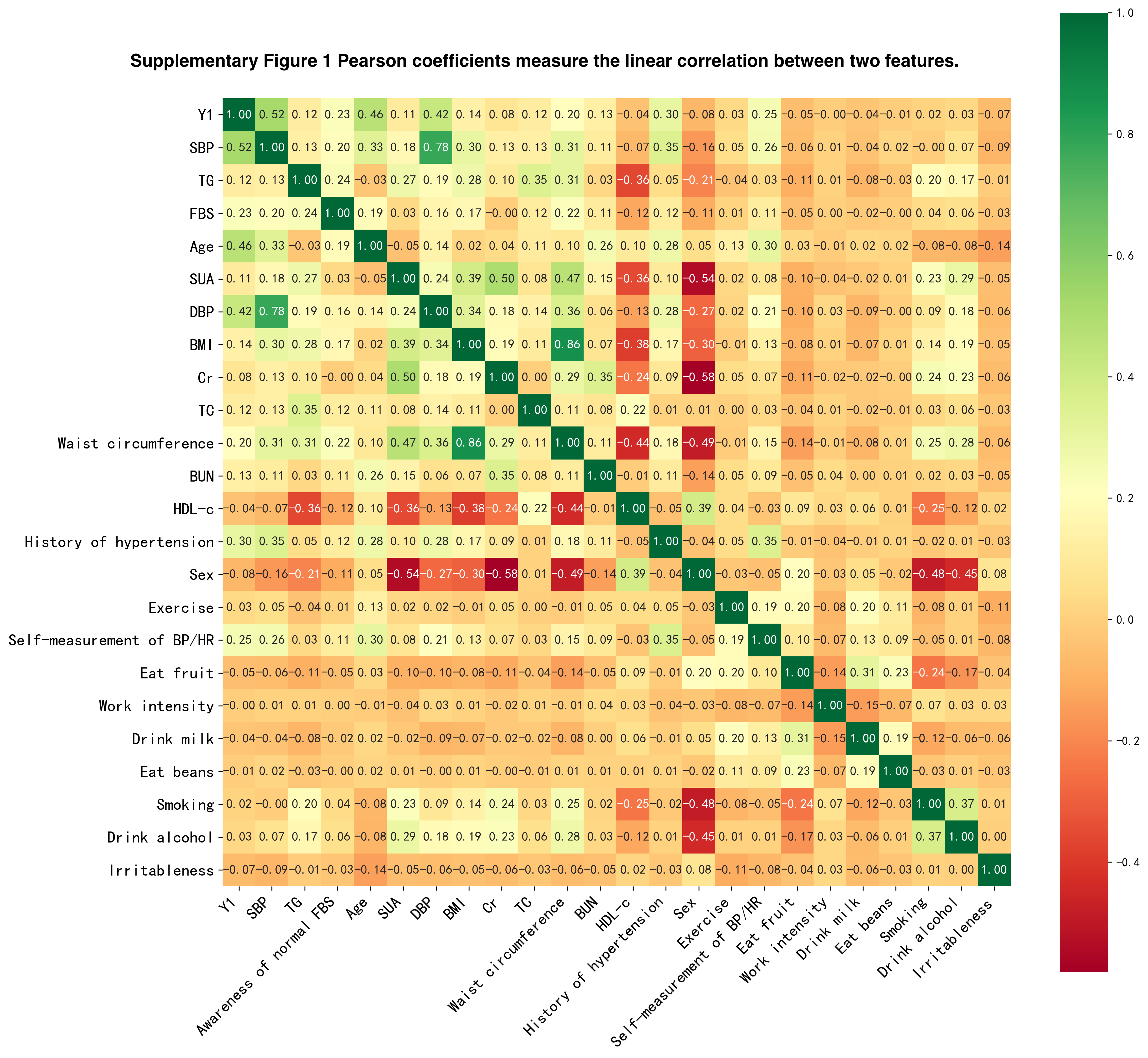

Supplement: Supplementary file 1 [file Image_1.JPEG]
