# Supplementary material for: A cost-effective, machine learning-driven approach for screening arterial functional aging in a large-scale Chinese population
Source: Front Public Health. 2024 Mar 20;12:1365479. doi: 10.3389/fpubh.2024.1365479 (PMC10987946; doi:10.3389/fpubh.2024.1365479)
Supplement: Supplementary file 2 [file Table_1.DOC]

**Supplementary Table 1** The results of classification algorithms based on 21 features including laboratory indicators.

| **model** | **accuracy _train** | **accuracy_test** | **sensitivity** | **specificity** | **AUC_test** |
| --- | --- | --- | --- | --- | --- |
| **LR** | 0.7890 | 0.7906 | 0.78 | 0.73 | 0.8746 |
| **RF** | 0.7872 | 0.7881 | 0.78 | 0.73 | 0.8697 |
| **XGB** | 0.7978 | 0.7912 | 0.79 | 0.73 | 0.8754 |
| **LGBM** | 0.7953 | 0.7900 | 0.78 | 0.73 | 0.8732 |

Footnotes: LR, logistic regression; RF, random forest; XGBoost, extreme gradient boosting; LightGBM, Light gradient boosting machine. RFE-LGBM, recursive feature elimination-Lightweight Gradient Elevator; AUC, area under the receiver operating characteristic curve.
